# Executive function and bilingualism in young and older adults

**DOI:** 10.3389/fnbeh.2014.00250

**Published:** 2014-07-25

**Authors:** Shanna Kousaie, Christine Sheppard, Maude Lemieux, Laura Monetta, Vanessa Taler

**Affiliations:** ^1^Bruyère Research Institute (Affiliated with the University of Ottawa)Ottawa, ON, Canada; ^2^Département de Réadaptation, Université LavalQuébec City, QC, Canada; ^3^Centre de Recherche de l’Institut Universitaire en Santé Mentale de QuébecQuébec City, QC, Canada; ^4^School of Psychology, University of OttawaOttawa, ON, Canada

**Keywords:** executive function, executive control, bilingualism, bilingual advantage, aging

## Abstract

Research suggests that being bilingual results in advantages on executive control processes and disadvantages on language tasks relative to monolinguals. Furthermore, the executive function advantage is thought to be larger in older than younger adults, suggesting that bilingualism may buffer against age-related changes in executive function. However, there are potential confounds in some of the previous research, as well as inconsistencies in the literature. The goal of the current investigation was to examine the presence of a bilingual advantage in executive control and a bilingual disadvantage on language tasks in the same sample of young and older monolingual anglophones, monolingual francophones, and French/English bilinguals. Participants completed a series of executive function tasks, including a Stroop task, a Simon task, a sustained attention to response task (SART), the Wisconsin Card Sort Test (WCST), and the digit span subtest of the Wechsler Adult Intelligence Scale, and language tasks, including the Boston Naming Test (BNT), and category and letter fluency. The results do not demonstrate an unequivocal advantage for bilinguals on executive function tasks and raise questions about the reliability, robustness and/or specificity of previous findings. The results also did not demonstrate a disadvantage for bilinguals on language tasks. Rather, they suggest that there may be an influence of the language environment. It is concluded that additional research is required to fully characterize any language group differences in both executive function and language tasks.

## Introduction

Executive functions, including inhibition, planning, and task switching, are important for everyday function. It is well established in the literature that normal aging is associated with changes in cognition, including executive functions, such as declines in inhibitory control (Hasher and Zacks, 1988) and processing speed (Salthouse, 1996), as well as language comprehension (Kemper, 2006). More recently, studies have shown that being bilingual may result in more efficient, resilient, and robust executive control processes, leading to superior performance on executive function tasks in bilinguals relative to monolinguals (see Bialystok et al., 2009; Adesope et al., 2010). Furthermore, these language group differences have been found to be larger in older than young adults (Bialystok et al., 2004), and it has been suggested that bilingualism may also delay the onset of Alzheimer’s disease symptoms (e.g., Bialystok et al., 2007, 2014; but see Chertkow et al., 2010; Zahodne et al., 2014). Given that over 50% of the world’s population is bilingual (Fabbro, 1999) and that older adults are the fastest growing demographic (Centers for Disease Control and Prevention, 2003; Statistics Canada, 2007) there are important implications for a “bilingual advantage” in executive function. The goal of the current investigation was to further examine the bilingual advantage in monolingual anglophones, monolingual francophones, and French/English bilingual young and older adults.

The bilingual advantage refers to findings demonstrating superior performance by bilinguals, relative to monolinguals, on tasks measuring inhibitory control. Specifically, advantages have been observed for bilinguals over monolinguals in interference suppression, which refers to the inhibition of task-irrelevant information, but not in response inhibition, which refers to the inhibition of a prepotent response (Bunge et al., 2002). These two components of inhibition can be differentiated using tasks such as the Stroop (1935) or Simon (Simon and Rudell, 1967) tasks to measure interference suppression, and the sustained attention to response task (SART; Robertson et al., 1997) to measure response inhibition. In the Stroop task an individual is required to inhibit the reading of a color word in order to correctly identify the (incongruent) color of the font that the word is printed in. For example, the word BLUE could be printed in blue ink on congruent trials and red ink on incongruent trials. The correct response would be “blue” and “red”, respectively; thus, on incongruent trials the participant would be required to inhibit the dominant word reading response in order to correctly identify the color of the ink. Stroop interference refers to the increase in response time (RT) for incongruent trials relative to neutral trials, where there is no color word information, or congruent trials. In one version of the Simon task the individual is required to ignore the spatial position of a stimulus and respond to some other dimension, such as the direction that an arrow is pointing in. For example, a left lateral key press in response to a leftward pointing arrow presented on the right of the screen would require the participant to ignore that the stimulus was presented on the right and respond only to the direction that the arrow is pointing in using a key on the left side of the keyboard. In contrast, the SART requires the participant to withhold a response to an infrequent stimulus, for example the number 3, within a string of stimuli that require a response, such as all other digits.

The bilingual advantage in interference suppression has been found in children (Bialystok and Martin, 2004), young adults (Bialystok et al., 2005; Costa et al., 2008) and older adults; the effect is the largest in the latter group (Bialystok et al., 2004). It is hypothesized that the constant management of two languages by bilinguals makes use of general executive control processes, for example, inhibiting one language while engaging in the other and effortlessly switching between languages when necessary (Bialystok, 2007, 2011; Bialystok et al., 2012). As a result, bilinguals receive extensive practice in these processes, and this experience is thought to be the mechanism underlying the observed bilingual advantage.

However, an advantage for bilinguals relative to monolinguals is not a consistent finding in the literature. Some research has found similar performance across language groups on executive function tasks (e.g., Kousaie and Phillips, 2012a,b; also see Paap and Greenberg, 2013). It is noteworthy that an advantage for bilinguals relative to monolinguals has also been found in working memory, albeit only for spatial material (Luo et al., 2013).

Interestingly, there is also a well-documented bilingual disadvantage on language tasks (see Michael and Gollan, 2005; Bialystok, 2009), including smaller vocabularies and difficulties with lexical access/retrieval (e.g., lower verbal fluency, more frequent tip-of-the-tongue states; longer picture naming latencies). For example, bilinguals have demonstrated a disadvantage relative to monolinguals on the Boston Naming Test (BNT; Kaplan et al., 1983; Gollan et al., 2007) which requires participants to name pictures that increase in difficulty as the task progresses, and working memory for verbal material (Luo et al., 2013). However, in the case of naming tasks, there exists some evidence that the bilingual disadvantage may have been overstated in the literature given that bilinguals have been found to show differential results depending on the method of scoring. Specifically, accepting responses in either language has been found to result in higher scores for bilinguals relative to an administration in which the bilinguals are required to respond in only one of their languages (Gollan et al., 2007).

Other researchers have examined language group differences in verbal fluency measures and found that bilinguals outperformed monolinguals on letter fluency, which has an executive component, but not on category fluency (Luo et al., 2010). Although, others have found a disadvantage for bilinguals on category fluency (Rosselli et al., 2000; Gollan et al., 2002), likely due to the reliance of the category fluency task on linguistic representations. It is noteworthy that Luo et al. subdivided their bilingual group based on vocabulary size, and bilinguals with a high vocabulary outperformed both monolinguals and bilinguals with a low vocabulary.

Taken together, the available evidence indicates that possessing mastery of two languages results in some advantages on executive control tasks and some disadvantages on language-specific tasks. However a number of issues have remained unaddressed in this literature, including whether observed advantages are confined to bilinguals who speak specific languages; whether there is a minimum level of proficiency/years of language experience required before an advantage emerges; and whether there is a particular language use profile that is necessary (e.g., one language at home, vs. another at school/work). Furthermore, the role of immigration status has not been fully explored. That is, in many of the studies that report advantages for bilinguals relative to monolinguals, a large proportion of the participants were immigrants who varied with respect to their native language (L1), or bilinguals who varied with respect to their second language (L2; Bialystok et al., 2006, 2008; Bialystok, 2006; Martin-Rhee and Bialystok, 2008; Luo et al., 2013). Given the many potential confounds that can be associated with participant characteristics such as immigration (e.g., diet, stress, life history; Chertkow et al., 2010), it is necessary to further examine the reported language group effects.

The goal of this investigation was to examine the bilingual advantage in executive function tasks and the bilingual disadvantage in language tasks in young and older monolingual francophones, monolingual anglophones, and French/English bilinguals in the same relatively well-controlled sample. We attempted to collect data from a comprehensive set of tasks measuring multiple aspects of executive function that have been used previously in the literature. We hypothesized that if there is in fact a robust bilingual advantage, bilinguals should outperform monolinguals on tasks of executive function. Specifically, bilinguals should show superior interference suppression relative to monolinguals (as measured by the Stroop and Simon tasks), but all three language groups should show similar response inhibition (as measured by the SART).

The consequences of bilingualism for working memory and cognitive flexibility are less clear. Given previous findings showing better spatial working memory for bilinguals than monolinguals (Luo et al., 2013) we expected to observe similar results for the digit span subtest of the Wechsler Adult Intelligence Scale (Wechsler, 1997). For the digit span task, participants are required to repeat lists of digits that increase in number first in the forward direction and then in the backward direction, starting with 2 digits and increasing by 1 to a maximum of 9 digits for forward digit span and 8 for backward digit span. We used the digit span task as a measure of working memory and expected that bilinguals would outperform monolinguals. To our knowledge, the only investigations to explore bilingualism and the Wisconsin Card Sorting Test (WCST), which measures cognitive flexibility and set-shifting, have examined bilinguals who frequently switch between their languages and those who do not (with non-switchers outperforming switchers; Festman and Münte, 2012) or compared monolinguals and bilinguals to simultaneous interpreters (with interpreters outperforming monolinguals and bilinguals, who did not differ; Yudes et al., 2011). For the WCST, participants are required to sort a set of cards based on a rule, color, shape or number, which switches following 10 consecutive correct responses. Participants are not informed of the rule they are supposed to use or when the rule switches, they are only given feedback on whether the current card was sorted correctly or not. Based on previous findings, we did not expect to see any clear differences between the language groups on the WCST.

Given previous findings demonstrating that the bilingual advantage is larger in older adults (Bialystok et al., 2004) we hypothesized that any observed language group effects would be larger in the older adults than in the younger adults. It is also possible that there could be significant language group differences in the older adults not observable in young adults given that the young adults are at the height of cognitive function and may not experience any additional benefit from being bilingual.

With respect to the language tasks, we expected that monolinguals would outperform bilinguals on the BNT, as has been found in previous studies (Gollan et al., 2007). Hypotheses regarding fluency tasks are less straightforward, given that these tasks also comprise an executive component; therefore, based on previous literature we tentatively hypothesized that bilinguals would outperform monolinguals on letter fluency given the executive demands required for this task, and that there would be no language group effect for category fluency (Luo et al., 2010) given the high level of proficiency of the bilinguals included in the present study. We included fluency measures for the letters F, A, and S, and for the category animals following Bialystok et al. (2008).

## Materials and methods

### Participants

Participants included monolingual and bilingual young (monolingual: *n* = 70; bilingual: *n* = 51) and older (monolingual: *n* = 61; bilingual: *n* = 36) non-immigrant adults recruited from the Ottawa and Quebec City communities. The monolingual young group comprised 30 French speakers and 40 English speakers, and the monolingual older group comprised 30 French speakers and 31 English speakers. Bilingual participants were relatively equally proficient in French and English, having self-reported high proficiency in their L2 before the age of 13 (see Table 1); proficiency in each language was determined using both self-report measures and an animacy judgement task described below (Segalowitz and Frenkiel-Fishman, 2005). Thirty-nine percent of young and 72 percent of older bilingual adults reported French as their native language, and the remainder reported English as their native language. Monolingual French speakers were recruited and tested in Quebec City, where the predominant language is French, while monolingual English speakers and bilinguals were recruited and tested in Ottawa, where the predominant languages are English and French. Within each age group, monolingual francophones, monolingual anglophones and bilinguals were matched for age, education, and general cognitive function as measured by the Montreal Cognitive Assessment (MoCA; Nasreddine et al., 2005), and over 90 percent of participants in each group were right handed. Monolinguals self-reported native-like ability in all aspects of their languages (i.e., reading, writing, speaking and listening) with minimal exposure to a second language, and bilinguals self-reported minimal exposure to any other languages besides English and French. Participant characteristics are provided in Tables 2 and 3.

**Table 1:** **Mean ranking (±standard deviation) for proficiency by modality for both L1 and L2 for bilingual participants**.

	**Young Adults (*n* = 51)**		**Older Adults (*n* = 36)**	
	**L1**	**L2**	**L1**	**L2**
Auditory Comprehension	5.00 ± 0.00	4.72 ± 0.50	4.97 ± 0.17	4.86 ± 0.42
Reading	4.94 ± 0.24	4.54 ± 0.65	4.92 ± 0.28	4.83 ± 0.38
Speaking	4.92 ± 0.27	4.40 ± 0.67	4.92 ± 0.28	4.83 ± 0.38
Writing	4.76 ± 0.47	4.15 ± 0.87	4.78 ± 0.48	4.69 ± 0.47

*Ranking followed a 5 point Likert scale (1 = no ability; 5 = native-like ability)*.

**Table 2:** **Young adult demographic, neuropsychological, executive function, and language task performance (mean ± standard deviation)**.

	**Anglophone** (***n* = 40; 15 males**)	**Francophone** (***n* = 30; 10 males**)	**Bilingual** (***n* = 51; 18 males**)
**Age** (years)	21.48 ± 1.50	21.80 ± 2.47	21.49 ± 2.26
**Education** (years)	15.55 ± 1.13	15.13 ± 1.38	15.49 ± 1.47
**Coefficient of Variablity**			
L1	0.21 ± 0.09	0.29 ± 0.14	0.27 ± 0.13
L2	NA	NA	0.28 ± 0.12
**MoCA**(/30)	28.60 ± 1.26	28.30 ± 1.73	28.04 ± 1.64
**WCST** (categories;/6)	4.40 ± 0.78	4.70 ± 0.47	4.47 ± 1.12
**Digit Span**			
Forward (/16)	10.98 ± 1.99	12.20 ± 2.19	11.41 ± 2.37
Reverse (/14)	6.98 ± 2.07	8.40 ± 2.66	7.80 ± 2.49
**SART**			
Reaction Time	348.01 ± 82.90	429.76 ± 129.87	354.90 ± 69.20
Errors	4.58 ± 3.38	3.45 ± 2.38	4.88 ± 4.39
**Simon Task**			
Control	400.09 ± 62.76	425.30 ± 103.18	396.64 ± 50.93
Reverse	503.26 ± 102.15	488.62 ± 96.97	491.24 ± 91.07
Conflict	561.21 ± 89.94	527.92 ± 48.26	549.65 ± 65.15
Congruent	558.21 ± 86.04	532.56 ± 44.92	549. 41 ± 62.65
Incongruent	564.59 ± 97.02	523.29 ± 53.75	549.93 ± 71.09
**Stroop**			
WR (/120)	110.13 ± 16.24	115.23 ± 15.86	105.02 ± 16.97
CN (/120)	78.85 ± 14.02	86.77 ± 14.37	76.39 ± 11.24
ICN (/120)	53.18 ± 12.02	48.67 ± 8.26	54.00 ± 11.07
WR-ICN	56.95 ± 10.77	66.57 ± 14.72	51.92 ± 19.00
CN-ICN	25.67 ± 7.00	38.1 ± 11.86	22.39 ± 9.53
**Verbal Fluency**			
FAS	39.95 ± 12.83	38.27 ± 8.26	37.24 ± 10.26
Animals	25.02 ± 5.90	23.00 ± 5.07	23.96 ± 5.77
**BNT**(/60)	53.20 ± 3.44	49.40 ± 4.55	46.92 ± 9.85

*ICN = incongruent colour naming; CN = colour naming; WR = word reading*.

**Table 3:** **Older adult demographic, neuropsychological, executive function, and language task performance (mean ± standard deviation)**.

	**Anglophone** (***n* = 31; 16 males**)	**Francophone** (***n* = 30; 7 males**)	**Bilingual** (***n* = 36; 19 males**)
**Age** (years)	72.26 ± 6.43	72.60 ± 6.59	70.69 ± 5.86
**Education** (years)	15.26 ± 2.87	16.20 ± 2.57	16.14 ± 2.85
**Coefficient of Variability**			
L1	0.25 ± 0.10	0.31 ± 0.15	0.27 ± 0.10
L2	NA	NA	0.27 ± 0.13
**MoCA**(/30)	27.94 ± 1.65	27.50 ± 1.36	27.53 ± 1.59
**WCST** (categories;/6)	3.50 ± 1.22	4.07 ± 0.69	3.50 ± 1.21
**Digit Span**			
Forward (/16)	11.19 ± 1.72	10.47 ± 2.40	10.28 ± 1.91
Reverse (/14)	7.87 ± 2.05	7.07 ± 2.03	7.06 ± 2.19
**SART**			
Reaction Time	461.17 ± 61.17	557.57 ± 140.20	482.90 ± 95.86
Errors	2.03 ± 1.89	1.10 ± 1.50	1.80 ± 1.83
**Simon Task**			
Control	622.55 ± 130.77	611.43 ± 152.17	634.20 ± 127.07
Reverse	885.03 ± 273.10	950.10 ± 313.48	901.08 ± 230.38
Conflict	799.79 ± 157.18	785.04 ± 190.32	797.12 ± 152.08
Congruent	775.25 ± 159.18	769.56 ± 182.05	777.78 ± 151.03
Incongruent	822.96 ± 162.23	800.91 ± 201.05	817.18 ± 156.48
**Stroop**			
WR (/120)	97.39 ± 12.96	104.07 ± 16.05	96.17 ± 15.34
CN (/120)	64.87 ± 11.73	73.10 ± 14.07	59.94 ± 12.57
ICN (/120)	34.77 ± 8.16	32.40 ± 9.55	36.72 ± 8.57
WR-ICN	62.61 ± 11.92	71.67 ± 14.80	59.44 ± 16.45
CN-ICN	30.10 ± 8.89	40.70 ± 12.05	23.22 ± 9.60
**Verbal Fluency**			
FAS	44.10 ± 10.00	36.37 ± 10.13	37.00 ± 13.26
Animals	20.68 ± 4.58	18.27 ± 4.62	19.17 ± 6.41
**BNT** (/60)	55.63 ± 3.16	46.93 ± 5.92	48.56 ± 6.00

*ICN = incongruent colour naming; CN = colour naming; WR = word reading*.

### Materials

#### Animacy judgement task

The animacy judgement task was used as an objective measure of relative second language (L2) proficiency and was based on the task used by Segalowitz and Frenkiel-Fishman (2005). Bilingual participants were presented with nouns on a computer monitor and were required to decide as quickly and accurately as possible whether each noun referred to something living or nonliving using the “1” and “2” keys on the keyboard. The task consisted of two separate language blocks, one in English, followed by one in French. Each comprised 64 trials (32 inanimate nouns and 32 animate nouns) and was preceded by eight practice trials. Monolingual anglophones and monolingual francophones completed only the English or French block respectively. The standard deviation for correct trials was divided by the RT for correct trials for each language block separately to obtain the coefficient of variability (CV), a measure of intraindividual variability in RT. The more similar the CV in a bilingual’s L1 and L2, the more relatively equally proficient the bilingual is believed to be (see Segalowitz and Segalowitz, 1993). Paired samples *t*-tests were used to compare the CVs in L1 and L2 for the bilingual young and older adults separately.

#### MoCA

The MoCA (Nasreddine et al., 2005) is a 12-min cognitive screening tool used to assess general cognitive function and detect mild cognitive impairment. The domains assessed include visuospatial and executive control, naming ability, memory, attention, language, abstraction, and orientation. The MoCA is scored out of 30 and a score of 26 or higher is considered normal. It was included here to ensure that all participants had normal cognitive functioning and that the language groups were matched on general cognitive function.^1^

#### Stroop task

The Stroop task (Stroop, 1935) was used as a measure of interference suppression. The version of the Stroop task used here included three conditions: word reading, color naming, and interference/incongruent color naming (naming the color of the print of incongruent color words; e.g., the word BLUE printed in red ink). For each condition, participants were presented with a sheet containing 4 columns of 30 stimuli appearing in random order and were asked to complete as many trials as possible in 45 s, starting with the first column and moving downward. In the word reading condition, the color words RED, GREEN, YELLOW, and BLUE were printed in black font and participants were asked to read as many words as possible. In the color naming condition, strings of six X’s were printed in either red, green, yellow, or blue font and participants were asked to name the color of as many of the stimuli as possible. In the incongruent condition, the color words RED, GREEN, YELLOW, and BLUE were printed in one of the incongruent colors, with each color-word combination appearing 10 times, and participants were asked to name the font color of as many words as possible without reading the word. All participants completed the word reading condition first, followed by the color naming condition, and the incongruent color naming condition was completed last. Anglophone and bilingual participants performed the task in English, while francophones performed it in French, and responses were recorded using Audacity 2.0 audio recorder and later played back to determine accuracy. The number of correct responses for each condition was counted.

#### Simon task

The Simon task (Simon and Rudell, 1967) was used as another measure of interference suppression. The version of the Simon task used here comprised three conditions: control, reverse, and conflict. In each condition, an arrow was presented on the monitor and participants were instructed to indicate, with the “A” and “L” keys on the keyboard, the direction of the arrow. In the control condition, the arrows appeared at the center of the monitor and participants were required to identify whether the arrow pointed to the left (by pressing the “A” key on the keyboard, located on the left side of the keyboard) or to the right (by pressing the “L” key on the keyboard, located on the right side of the keyboard). In the reverse condition, the arrows appeared at the center of the screen and the participant was required to identify the direction of the arrow using the key on the opposite side on the keyboard; i.e., “A” for a rightward pointing arrow and “L” for a leftward pointing arrow. In the conflict condition, the arrows were presented on either the left or right side of the monitor, creating congruent (e.g., rightward pointing arrow presented on the right) and incongruent trials (e.g., leftward pointing arrow presented on the right). For both the control and reverse condition, there were two blocks of 48 trials each. For the conflict condition, there were a total of 192 trials split into two blocks, with 48 congruent and 48 incongruent trials in each block. At the beginning of each new condition there was a series of practice trials (one practice trial for each trial type); the order of presentation of the conditions was counterbalanced across participants, and stimuli were presented in randomized order within each condition.

#### SART

The SART (Robertson et al., 1997) was used as a measure of response inhibition. For this task, participants were presented with the digits 1 through 9 on the computer screen and were required to press the space bar in response to every number except the number 3, for which no response was required. There were 25 blocks of nine trials, and each number appeared once in each block. The numbers were randomized within each block and the participants were not informed of the number of blocks that they would be completing, or the number of “3”s that would appear in each block. Each trial was preceded by a mask (######) that appeared for 500 ms, and the participant’s response initiated the subsequent trial, except when the stimulus was the number 3, which stayed on the screen for 2000 ms.

#### Digit span

The forward and backward digit span subtests of the Wechsler Adult Intelligence Scale III (Wechsler, 1997) were administered as a measure of working memory. In this task, the experimenter read the participant a list of digits that the participant was asked to repeat in either the forward or backward order, depending on the task. The list started with 2 digits and the span increased by 1 digit until a maximum of 9 for the forward and 8 for the backward digit span tasks. There were two trials at each span length resulting in a maximum possible score of 16 for forward digit span and 14 for backward digit span, and the task was discontinued when the participant made an error on both trials at any span length.

#### WCST

The WCST (Grant and Berg, 1948) measures set-shifting/ cognitive flexibility, and a participant’s ability to adapt to changing demands and schedules of reinforcement. In this task participants are asked to sort a series of 64 cards based on three possible criteria: color, shape/form, and number. Four cards (one with a single red triangle, the second with 2 green stars, the third with 3 yellow “+” signs, and the fourth with 4 blue circles) are laid down in front of the participant. Participants are instructed to sort the cards (each containing 1–4 of the above-mentioned shapes in any of the four colors) into piles according to the four cards placed in front of them, whereupon the experimenter informs the participant whether the card was sorted correctly or incorrectly. The sorting rule changes each time the participant correctly categorizes 10 consecutive cards; the sorting rule begins as “color”, then switches to shape/form, and then to number, and then repeats in this order, following standardized instructions, until all the cards have been sorted. A point is awarded each time the participant achieves a category (i.e., 10 consecutive correct responses), resulting in a maximum score of 6.

#### BNT

The BNT (Kaplan et al., 1983) is a picture naming task comprising 60 images. Participants are presented with the images one at a time and are asked to name them. BNT performance is used to measure language function and can be used to help diagnose cognitive status (e.g., Mungas et al., 2005). Standardized scoring procedures were used; one point was awarded for each correctly identified image, if a stimulus cue was needed one point was awarded if the pictures was correctly identified following the semantic cue, but not following the phonemic cue. Bilingual participants completed the BNT three times: once in French, once in English, and once in a condition where they could respond in either language (bilingual administration) in randomized order; the data from their L1 are reported here.

#### Verbal fluency

Participants completed letter fluency for the letters F, A, and S and category fluency for the category animals (Controlled Oral Word Association test; Benton and Hamsher, 1976). In bilinguals this was done in both French and English, as well a condition in which they could respond in either language, in randomized order; the data from their L1 are reported here. In the letter fluency task, participants are asked to generate as many words as possible in 1 min, beginning with the specified letter. The total number of words generated was counted, excluding repetitions, numbers, proper nouns and words of the same root (e.g., love, lover, loving). In the category fluency task, participants name as many animals as they can in 1 min. The total number of words generated was counted, excluding repetitions. Responses were recorded using Audacity 2.0 audio recorder^2^ and transcribed later.

### Apparatus

Several tasks were completed on a laptop computer, including the animacy judgement task, the Simon task, and the SART. Regardless of the task, stimuli were presented using E-Prime 2.0 presentation software (Psychology Software Tools, Pittsburg, PA, USA); however, three different laptops were used to collect the data. At the Quebec City site, the data were collected using a Toshiba Portégé A600 laptop with a 12.1″ screen, Windows 7 operating system and an Intel Centrino 2 processor (all monolingual francophone participants were tested using this hardware). At the Ottawa site, the majority of the participants were tested using a Dell Inspiron Mini with a 10″ screen, Windows XP operating system and Intel Atom processor. However, one monolingual and two bilingual young adults were tested using a Dell Latitude E4310 laptop with a 12.1″ screen, Windows XP operating system and Intel Core i5 processor.

Given that the data were collected using different hardware, several additional analyses were conducted to ensure that there were no systematic differences in the data collected from the different laptops. We conducted an independent samples *t*-test, for the young and older adults separately, comparing the data from monolingual francophones (tested using the Portégé A600 laptop) and monolingual anglophones (tested using Dell Inspiron Mini laptop) for the Simon and SART tasks. These analyses showed that there were no RT differences in the data for either age group on any of the conditions of the Simon task (control, reverse, and conflict conditions; all *p*s > 0.08); however, monolingual francophones showed longer RTs for the SART than monolingual anglophones (*M* = 80.1 ms for the young adults and 96.4 ms for the older adults). Given that only one monolingual and two bilingual young adults were tested using the Dell Latitude laptop there were not enough data to run a valid *t*-test to compare the data collected using these different laptops. Following these additional analyses we were confident that combining the data collected with different hardware would not introduce any confounds, except perhaps in the case of the SART.

### Procedure

Data from monolingual anglophones and bilinguals for the current investigation were collected as part of a larger study. Therefore, participants visited the laboratory on two occasions each lasting between 1.5 and 2 h. Informed consent was obtained and participants completed a series of paper-and-pencil and computerized tasks, including those reported here. At the end of the second session, participants were debriefed and compensated $10 per hour of participation. This study was approved by the Research Ethics Board at the Bruyère Research Institute and the University of Ottawa.

Data from monolingual francophones were collected in two sessions, each lasting 1 h. E-Prime data (i.e., Animacy judgement, Simon and SART) were collected during the second session. Informed consent was obtained at the beginning of the testing session. At the end of the session participants were debriefed and compensated at the rate of $10 per hour of participation. This study was approved by the Research Ethics Board at Laval University.

All participants followed the same procedure, independent of testing site. Tasks were administered in the following order for the monolinguals: MoCA, verbal fluency and BNT, Simon task, animacy judgement task, Stroop task, WCST, digit span, SART. For bilinguals the first session included the MoCA, verbal fluency and BNT (English, French or bilingual administration), Simon task, animacy judgement task, verbal fluency and BNT (English, French or bilingual administration), and the second session included verbal fluency and BNT (English, French or bilingual administration), Stroop task, WCST, digit span, and SART, administered in that order. The different language administrations were randomized across bilingual participants.

## Results

All statistical analyses were conducted using PASW Statistics 18 using an *α*-level of 0.05, unless otherwise specified. All RT data trials for which RTs were greater than ±2.5 standard deviations from the mean were excluded as outliers by participant and condition. Unless otherwise specified, we conducted an analysis of variance (ANOVA) for each of the tasks comparing young and older adults (Age Group), and monolingual francophones, monolingual anglophones, and bilinguals (Language Group). We report all significant main effects and interactions; any significant interactions were followed up with simple effects analyses. All the data are presented in Tables 1 and 2. Given that Language Group effects were of primary interest in the current investigation, these effects are summarized for the executive function and language tasks in Table 4. Technical difficulties resulted in the loss of a small portion of data; given that this was not consistent across tasks, any missing data is reported separately for each task.

**Table 4:** **Summary of language group (and language group by age interaction) effects by task**.

**Task**	**Summary of language group effects**
**Executive Function tasks**	It was hypothesized that bilinguals would show superior performance on these tasks relative to monolinguals
Stroop task (number of correct responses)	• BI < MF overall • WR & CN: MA, BI < MF; ICN: MF < MA, BI
Stroop interference (difference in number of correct responses)	• *WR-ICN* : MF > MA, BI • *CN-ICN* : MF > MA > BI
Simon task (RT)	NO EFFECT OF LANGUAGE GROUP
Simon interference (RT for incongruent—RT for congruent)	• MA > MF
SART (RT)	• MF > MA, BI
Digit span forward (number of correct responses)	• MF & BI: older < young; MA: older = young
Digit span backward (number of correct responses)	• MF: older < young; MA, BI: older = young
WCST (number of categories achieved)	• MF > MA, BI
**Language Tasks**	It was hypothesized that monolinguals would outperform bilinguals on these tasks
BNT (number of correct items)	• MF, BI < MA
Letter fluency (number of correct items produced)	• MF, BI < MA
Category fluency (number of correct items produced)	• MF < MA

*BI = bilingual; MF = monolingual francophones; MA = monolingual anglophones; ICN = incongruent colour naming; CN = colour naming; WR = word reading*.

### Animacy judgement task

Data were missing for two young bilinguals. The CV was calculated by dividing the standard deviation for correct trials by the mean RT for correct trials for each participant and language separately. We conducted separate paired samples *t*-tests for the young and older bilingual adults in order to compare the CVs in L1 and L2 and ensure that participants were relatively equally proficient in both of their languages. The *t*-tests revealed no significant difference in the CVs between the L1 and L2 for the young (*t*_(48)_ = −0.42, *p* = 0.67) or the older (*t*_(35)_ = −1.1, *p* = 0.28) adults, suggesting that bilingual participants were highly proficient in their L2.

### Executive function tasks

#### Stroop task

Data were missing for one young anglophone and three young bilinguals. The repeated measures ANOVA including the within-subjects factor Condition (word reading, color naming, incongruent color naming) revealed main effects of Age Group (*F*_(2,208)_ = 95.93, *MSE* = 324.88, *p* < 0.01, $\eta_{p}^{2}$ = 0.32), showing that older adults generated fewer responses than young adults; Language Group (*F*_(2,208)_ = 5.02, *MSE* = 324.88, *p* < 0.01, $\eta_{p}^{2}$ = 0.05), showing that bilinguals generated fewer correct responses than monolingual francophones; and Condition (*F*_(2,416)_ = 2236.35, *MSE* = 87.98, *p* < 0.01, $\eta_{p}^{2}$ = 0.92, showing a difference between all three conditions, with the highest number of correct responses for word reading and the fewest for incongruent color naming. In addition, there was a Language Group × Condition interaction (*F*_(4,416)_ = 15.45, *MSE* = 87.98, *p* < 0.01, $\eta_{p}^{2}$ = 0.13), showing that monolingual francophones generated more correct responses than monolingual anglophones and bilinguals for word reading and color naming (all *p*s < 0.05), but fewer correct incongruent color naming responses than both monolingual anglophones (*p* = 0.04) and bilinguals (*p* < 0.01).

In addition, we analyzed language group differences in two different measures of Stroop interference (i.e., the decrease in correct response for incongruent color naming relative to a neutral condition). Interference was calculated relative to both word reading and color naming by subtracting the number of correct responses for the incongruent color naming condition from the number of correct responses for the word reading condition and from the color naming condition, respectively. Young adults showed less interference than older adults when interference was calculated relative to both word reading (*F*_(1,208)_ = 8.42, *MSE* = 229.58, *p* < 0.01, $\eta_{p}^{2}$ = 0.04) and color naming (*F*_(1,209)_ = 3.72, *MSE* = 96.18, *p* = 0.055, $\eta_{p}^{2}$ = 0.02). In terms of language group differences, monolingual francophones showed greater interference than monolingual anglophones and bilinguals when interference was relative to word reading (*F*_(2,208)_ = 13.89, *MSE* = 229.58, *p* < 0.01, $\eta_{p}^{2}$ = 0.12). When interference was relative to color naming, however, all three language groups differed, with the least interference demonstrated by bilinguals and the most by monolingual francophones (*F*_(2,209)_ = 50.72, *MSE* = 96.18, *p* < 0.01, $\eta_{p}^{2}$ = 0.33).

#### Simon task

Data were missing for 1 young and 1 older francophone, 1 older anglophone, and 1 young and 3 older bilinguals. The data for conditions with central (control and reverse) and lateral (conflict) presentation were analyzed separately. A repeated measures ANOVA including the within-subjects factor Condition (control vs. reverse) revealed faster responses for young than older adults (main effect of Age Group, *F*_(1,204)_ = 260.7, *MSE* = 38681.4, *p* < 0.01, $\eta_{p}^{2}$ = 0.56). We also found a main effect of Condition *F*_(1,204)_ = 334.3, *MSE* = 10625.5, *p* < 0.01, $\eta_{p}^{2}$ = 0.62), whereby responses were faster in the control than reverse condition. Finally, an Age × Condition interaction (*F*_(1,204)_ = 95.9, *MSE* = 10625.5, *p* < 0.01, $\eta_{p}^{2}$ = 0.32) showed that the increase in RT for the reverse relative to the control condition was larger in older than young adults (87.03 vs. 287.84 ms). A second repeated measures ANOVA was conducted to compare congruent and incongruent trials in the conflict condition. There were main effects of Age (*F*_(1,205)_ = 205.8, *MSE* = 30307.5, *p* < 0.01, $\eta_{p}^{2}$ = 0.50) and Trial Type (*F*_(1,205)_ = 58.2, *MSE* = 654.5, *p* < 0.01, $\eta_{p}^{2}$ = 0.22) showing faster responses for young adults and congruent trials relative to older adults and incongruent trials, respectively. There was also a significant interaction between Age and Trial Type (*F*_(1,205)_ = 63.0, *MSE* = 654.5, *p* < 0.01, $\eta_{p}^{2}$ = 0.24), demonstrating that only the older adults showed an increase in RT for incongruent relative to congruent trials (39.49 ms).

Of critical interest in this task was Language Group differences in interference suppression. We subtracted the RT for congruent trials from the RT for incongruent trials within the conflict condition to obtain an interference score and conducted a one-way ANOVA on these scores with Language Group and Age Group as the between subjects factors. Overall, older adults showed larger interference effects than younger adults (main effect of Age Group (*F*_(1,205)_ = 63.04, *MSE* = 1309.02, *p* < 0.01, $\eta_{p}^{2}$ = 0.24), and monolingual anglophones showed larger interference effects than monolingual francophones (main effect of Language Group (*F*_(2,205)_ = 3.09, *MSE* = 1309.02, *p* = 0.05, $\eta_{p}^{2}$ = 0.03).

#### SART

Analysis of data from the SART revealed a main effect of Age Group (*F*_(1,204)_ = 81.34, *MSE* = 9458.51, *p* < 0.01, $\eta_{p}^{2}$ = 0.29), whereby older adults responded more slowly than young adults. We also found a main effect of Language Group (*F*_(2,204)_ = 15.0, *MSE* = 9458.51, *p* < 0.01, $\eta_{p}^{2}$ = 0.13), whereby RTs were longer in monolingual francophones than in monolingual anglophones and bilinguals. Moreover, older adults committed fewer errors than young adults (*F*_(1,204)_ = 41.27, *MSE* = 8.69, *p* < 0.01, $\eta_{p}^{2}$ = 0.17).

#### Digit span

A separate ANOVA was conducted for the forward and backward digit span tasks. There was a main effect of Age Group on the forward digit span (*F*_(1,212)_ = 9.07, *MSE* = 4.52, *p* < 0.01, $\eta_{p}^{2}$ = 0.04), showing that the young adults achieved higher scores than the older adults. However, an Age Group × Language Group interaction (*F*_(2,212)_ = 3.69, *MSE* = 4.52, *p* = 0.03, $\eta_{p}^{2}$ = 0.03) demonstrated that young adults achieved higher scores than older adults in the monolingual francophone (*p* < 0.01) and bilingual groups only (*p* = 0.02), whereas there was no effect of Age Group in monolingual Anglophones (*p* = 0.67).

Analysis of the backward digit span revealed an Age Group × Language Group interaction (*F*_(2,212)_ = 4.35, *MSE* = 5.17, *p* = 0.01, $\eta_{p}^{2}$ = 0.04), whereby young monolingual francophones achieved higher scores than older monolingual francophones (*p* = 0.02). The simple effect of Age Group was not significant in monolingual anglophones (*p* = 0.10) or bilinguals (*p* = 0.13).

#### WCST

Analysis of the WCST revealed that young adults obtained more categories than older adults (main effect of Age Group (*F*_(1,209)_ = 38.09, *MSE* = 0.95, *p* < 0.01, $\eta_{p}^{2}$ = 0.15), and monolingual francophones achieved more categories than monolingual anglophones and bilinguals (main effect of Language group (*F*_(2,209)_ = 3.91, *MSE* = 0.95, *p* = 0.02, $\eta_{p}^{2}$ = 0.04).

### Language tasks

For these analyses only data from the bilinguals’ L1 were included.

#### BNT

Analysis of the BNT revealed a main effect of Language Group (*F*_(2,209)_ = 24.77, *MSE* = 39.65, *p* < 0.01, $\eta_{p}^{2}$ = 0.19), whereby anglophones obtained more correct responses than francophones and bilinguals.

#### Verbal fluency

The total number of words generated for each of the letter fluency tasks (F, A, and S) was summed to obtain a single score for each participant. The analysis revealed that monolingual anglophones generated more words than monolingual francophones and bilinguals (main effect of Language Group *F*_(2,210)_ = 4.47, *MSE* = 121.2, *p* = 0.01, $\eta_{p}^{2}$ = 0.04). In category fluency young adults generated more animal names than older adults (main effect of Age Group *F*_(1,210)_ = 37.66, *MSE* = 30.36, *p* < 0.01, $\eta_{p}^{2}$ = 0.15). There was also a trend toward a main effect of Language Group (*F*_(2,210)_ = 2.90, *MSE* = 30.36, *p* = 0.06, $\eta_{p}^{2}$ = 0.03), whereby monolingual anglophones generated more animal names than monolingual francophones.

## Discussion

The goal of the current investigation was to further examine language group differences in executive function and language tasks in a group of young and older monolingual francophones, monolingual anglophones and French/English bilinguals. Previous research has found that bilinguals demonstrate advantages on tasks of executive function and disadvantages on language tasks, relative to monolinguals. A larger advantage on executive function tasks has been reported in older adults relative to young adults. However, questions arise with respect to some socio-demographic variables (e.g., immigration status) of some of the samples studied in previous research. Therefore, in the current investigation we controlled for immigration status and languages spoken. We hypothesized that, if there is a robust executive function advantage for bilinguals, it should emerge in our data, and it should be larger in older than younger adults. We also expected to replicate previous findings showing disadvantages for bilinguals on language tasks.

Measures of interference suppression (Stroop and Simon tasks), response inhibition (SART), working memory (forward and backward digit span) and cognitive flexibility (WCST) were used to assess executive function. Our hypotheses regarding interference suppression and response inhibition were clear: bilinguals should show better performance and less interference than monolinguals on the Stroop and Simon tasks, and all groups should perform similarly on the SART. In general, these hypotheses were not supported.

Specifically, in the Stroop task, there was weak support for a bilingual advantage in that monolingual francophones produced fewer incongruent color naming responses than bilinguals (and monolingual anglophones, who did not differ from bilinguals). Monolingual francophones also exhibited greater interference (i.e., greater decrease in the number of correct responses for incongruent color naming relative to a neutral condition) than bilinguals, regardless of how the interference score was computed (i.e,. relative to word reading or color naming). Monolingual anglophones also demonstrated more interference than bilinguals (but less than monolingual francophones), but only when interference was calculated relative to color naming. For the Simon task, there were no language group effects for the raw RT data. Furthermore, in comparison with either monolingual group, bilinguals did not show smaller interference, despite monolingual anglophones showing larger interference than monolingual francophones. Given that both the Simon task and the Stroop task measure interference suppression, it is interesting that the two tasks result in contrasting findings. It is unclear why this is the case; one possibility is that it is due to the Stroop task having a language component. Finally, monolingual francophones showed longer RTs for the SART than monolingual anglophones and bilinguals, who did not differ from each other. The hypotheses regarding working memory and cognitive flexibility were less straightforward. The results showed that there were no differences between monolinguals and bilinguals for the forward or backward digit span, and monolingual francophones outperformed monolingual anglophones and bilinguals (who did not differ) on the WCST.

The results of the executive function tasks do not provide clear evidence for a bilingual advantage and the findings are not consistent across the tasks. For Simon interference and cognitive flexibility, monolingual francophones show an advantage relative to both monolingual anglophones and bilinguals; for the Stroop task and response inhibition, in contrast, monolingual francophones show a disadvantage relative to the two other groups. It is important to note that there was a significant difference in RT for the SART between monolingual anglophones and francophones who were tested using different laptop computers; language group differences for response inhibition may thus be due to the use of different testing equipment rather than a true language group effect.^3^ The only result supporting a purely bilingual advantage was for Stroop interference, where bilinguals showed smaller interference relative to both monolingual groups.

These findings suggest that the language group differences observed here are the result of something other than bilingualism. The two monolingual groups were tested in different locations, therefore complicating interpretation of the results; however, data that were collected using different computers were compared and found to be similar, with the exception of the SART. Therefore, the results suggest that there may be a cultural effect driving the observed language group differences. This is an interesting possibility given that French is the predominant language in Quebec City, whereas English and French are both commonly used in the city of Ottawa. This implies that the English monolinguals included here may have been exposed to French on a more regular basis whereas the French monolinguals are not exposed to English with the same frequency. However, critically, monolingual anglophones and bilinguals, who were living in and tested in the same location (i.e., Ottawa, Ontario), did not differ on any of the tasks except Stroop interference. Furthermore, there were no instances in which any language group differences were larger for older than young adults.

In order to assess any disadvantages in language performance, the BNT, letter and category fluency tasks were included in the test battery. It was hypothesized that monolinguals would show an advantage for the BNT; however, the results indicate an advantage only for monolingual anglophones relative to monolingual francophones and bilinguals, which partially supports our hypothesis. It is unclear why only the anglophones show an advantage relative to the bilinguals and perform better than the francophones; this may suggest that the BNT is more difficult in French than in English, or that the items are less prototypical in French culture, resulting in a familiarity effect. Although there is no empirical evidence to support the claim that the BNT is more difficult in French, there is some evidence that the difficulty of the BNT varies based on language background and the languages that an individual knows (Roberts et al., 2002; Rosselli et al., 2012). It is noteworthy that Roberts et al. included French Canadians in their investigation, and French-English bilinguals performed worse than English monolinguals.

The results of the fluency tasks do not support our hypotheses: monolingual anglophones produced more correct responses than monolingual francophones on the category fluency task, while in the letter fluency task, monolingual anglophones outperformed both monolingual francophones and bilinguals. The scoring method that was used entailed combining the scores to obtain a composite score for the three letter fluency tasks, as has been done in previous investigations (Bialystok et al., 2008). It is possible that an alternative scoring method, such as examining clustering (i.e., generating similar items close together in sequence) and switching (i.e., shifting from one subcategory to another; Troyer et al., 1997) would reveal language group differences; this possibility should be explored in future research.

Taken together, the results of the executive function and language tasks raise questions about the reliability, robustness, and specificity of the purported “bilingual advantage”. There are several possible reasons why the data reported here do not support previous findings, all of which imply that the bilingual advantage may be less robust, or more specific than previously suggested. One such explanation is that in addition to being non-immigrants, bilingual participants in this study likely have a very different language-use profile than bilinguals included in other studies. That is, the language environment of bilingual participants included here exposes them to both of their languages on a very regular and consistent basis in most situations that they encounter, given the bilingual nature of the city of Ottawa. This language use/exposure differs from that of many other bilinguals who vary with respect to their two languages and may use each of their languages in very specific and separate situations (e.g., one language at home, the other language at school/work). It is possible that these language-use differences affect the cognitive consequences of bilingualism. Recently, there has been substantial interest in codeswitching and how this behavior may lead to different cognitive outcomes. For example, Festman and Münte (2012) found that individuals who exhibited cross-language interference on a bilingual picture naming task (i.e., language switchers) performed worse than non-switchers on the WCST and a flanker task.

It is also possible that our measures of interference suppression were not sensitive enough to detect differences between monolinguals and bilinguals, particularly in the young adults. That is, language group differences in young adults who are at the height of cognitive function may be more subtle and difficult to detect. This is supported by previous research that has found language group differences in brain-based measures, but no differences in behavior (e.g., Bialystok et al., 2005; Kousaie and Phillips, 2012b). However, the current results do show canonical interference and age effects, suggesting that the tasks themselves were effective at introducing interference. Another possible explanation related to task sensitivity is that the tasks were too long, allowing monolinguals enough practice to overcome any initial disadvantage relative to bilinguals. Previous research has found that over the course of multiple blocks of trials there is convergence between the performance of monolinguals and bilinguals (Bialystok et al., 2004; also see Hilchey and Klein, 2011). In order to address this possibility we conducted a supplemental analysis of the Simon interference effect including the first block of trials only. This analysis revealed similar findings as the overall interference analysis, indicating that the lack of a bilingual advantage in Simon interference is unlikely to be the result of too many trials. Unfortunately, given the nature of the Stroop task it was not possible to examine any practice effects on Stroop interference. Finally, our tasks may not have been challenging enough—others have found that the bilingual advantage only emerges under conditions that are demanding of monitoring processes (e.g., Bialystok et al., 2004; Bialystok, 2006; Costa et al., 2009).

Interestingly, there was some evidence of a monolingual francophone advantage in Stroop word reading and color naming, Simon interference, and the WCST. As previously mentioned, given the possibility of confounds resulting from different testing environments, this finding is difficult to interpret. However, it may suggest the existence of effects associated with the specific language(s) that an individual speaks. Alternatively, the specific environment in which an individual lives may exert an effect; for example, in Quebec City the predominant language is French, and monolinguals are likely exposed to English much less frequently than anglophones in the bilingual city of Ottawa are to French. This finding merits further investigation; future research should compare monolingual francophones and French/English bilinguals in the same location and testing environment.

In conclusion, the current investigation does not provide convincing support for a bilingual advantage on executive function tasks and does not replicate previous findings of a bilingual disadvantage for language tasks. Our conclusions are tentative given the difficulties associated with interpreting null results; however, the data presented here raise questions with respect to the robustness, reliability and specificity of such an advantage. Despite the limitations of this study (e.g., two groups of monolinguals from different locations) it is clear that additional research is required to fully characterize both the potential advantages and disadvantages associated with being bilingual. Given the importance of executive function and language-based tasks for neuropsychological assessment, this area of research has important clinical implications and it is imperative that we understand the consequences of bilingualism on the performance of these tasks. In terms of broader implications, the current investigation demonstrates the importance of context to the development of executive function processes, particularly language context. Given the influence of highly plastic language functions on executive function processes, an argument can be made for the utility of other cognitive training programs for the recovery of executive function in populations experiencing deficits resulting from age-related decline and/or neuropathology.

## Conflict of interest statement

The authors declare that the research was conducted in the absence of any commercial or financial relationships that could be construed as a potential conflict of interest.

## Footnotes

^1^Note that 1 monolingual anglophone, 1 monolingual francophone and 4 bilingual young adults, and 2 monolingual anglophone and 4 bilingual older adults obtained scores of 24 or 25 on the MoCA; however, based on their interactions with the experimenter and performance on other neuropsychological assessments they were deemed to have normal cognitive function. Critically, within each age group the language groups were matched for MoCA score.

^2^http://audacity.sourceforge.net

^3^The independent samples *t*-tests that we conducted indicated that the use of different testing computers across sites did not result in systematic differences in RT data for the Simon task, despite differences for the SART task. Given that the critical test of the bilingual advantage is language group differences in Simon interference the use of different computers poses less of a risk in terms of our conclusions for the Simon task given that any effect of hardware would be similar across conditions.
